# Functional polymorphisms in genes of the *Angiotensin *and *Serotonin *systems and risk of hypertrophic cardiomyopathy: *AT1R *as a potential modifier

**DOI:** 10.1186/1479-5876-8-64

**Published:** 2010-07-01

**Authors:** Eliecer Coto, María Palacín, María Martín, Mónica G Castro, Julián R Reguero, Cristina García, José R Berrazueta, César Morís, Blanca Morales, Francisco Ortega, Ana I Corao, Marta Díaz, Beatriz Tavira, Victoria Alvarez

**Affiliations:** 1 Genética Molecular, Red de Investigación Renal (REDINREN), and Fundación Renal; Hospital Universitario Central de Asturias; Oviedo; Spain; 2 Servicio de Cardiología, Fundación ASTURCOR; Hospital Universitario Central de Asturias; Oviedo; Spain; 3 Servicio de Cardiología, Hospital Universitario M. Valdecilla; Santander; Spain; 4 Servicio de Nefrología, Red de Investigación Renal (REDINREN), and Fundación Renal; Hospital Universitario Central de Asturias; Oviedo; Spain; 5 Departamento de Medicina, Universidad Oviedo; Oviedo; Spain

## Abstract

**Background:**

Angiotensin and serotonin have been identified as inducers of cardiac hypertrophy. DNA polymorphisms at the genes encoding components of the angiotensin and serotonin systems have been associated with the risk of developing cardiovascular diseases, including left ventricular hypertrophy (LVH).

**Methods:**

We genotyped five polymorphisms of the *AGT*, *ACE*, *AT1R*, *5-HT2A*, and *5-HTT *genes in 245 patients with Hypertrophic Cardiomyopathy (HCM; 205 without an identified sarcomeric gene mutation), in 145 patients with LVH secondary to hypertension, and 300 healthy controls.

**Results:**

We found a significantly higher frequency of *AT1R *1166 C carriers (CC+AC) among the HCM patients without sarcomeric mutations compared to controls (p = 0.015; OR = 1.56; 95%CI = 1.09-2.23). The *AT1R *1166 C was also more frequent among patients who had at least one affected relative, compared to sporadic cases. This allele was also associated with higher left ventricular wall thickness in both, HCM patients with and without sarcomeric mutations.

**Conclusions:**

The 1166 C *AT1R *allele could be a risk factor for cardiac hypertrophy in patients without sarcomeric mutations. Other variants at the *AGT*, *ACE*, *5-HT2A *and *5-HTT *did not contribute to the risk of cardiac hypertrophy.

## Introduction

Left-ventricular hypertophy (LVH) is a physiological adaptation of the heart to increased workload. LVH is frequently secondary to clinical conditions such as hypertension, valvular disease, and myocardial infarction [1,2]. However, some patients develop the cardiac hypertrophy in the absence of these conditions that impose overwork to the heart. This primary/essential form of LVH is frequently familial and caused by mutations in sarcomeric genes, and is designated as hypertrophic cardiomyopathy (HCM) [3]. Some patients with HCM lack a family history of the disease, and are thus regarded as sporadic cases. Several gene polymorphisms have been associated with the risk of developing LVH, and could also modify the clinical phenotype in HCM patients [4-6]. Neurohumoral factors such as angiotensin II (Ang) and serotonin (5-hydroxytriptamine; 5-HT) have been identified as inducers of cardiac hypertrophy [7,8]. These molecules bind to G protein-coupled receptors on cardiac fibroblasts, and stimulate the production and release of growth factors and cytokines that would induce cardiomyocyte hypertrophy [9,10]. The interactions between the angiotensin and serotonin systems in cardiac cells could play a major role in the development of cardiac hypertrophy [8].

Serotonin is a molecule produced by several cell types, such as serotonergic neurons and renal proximal tubular cells. A large amount of serotonin is stored in blood platelets, bounded to the serotonin transporter (5-HTT). This serotonin is released during platelet activation and binds to specific receptors on target cells stimulating a wide array of physiological changes, such as platelet aggregation, vascular contraction, and hyperplasia of the smooth muscle cells [11,12]. In the heart, serotonin stimulates sympathetic afferent nerves and causes contraction of the coronaries during ischemia. Studies with mice genetically modified for 5-HT receptors implicated the serotonin pathway in ventricular hypertrophy [13,14]. This pro-hypertrophic effect would require the uptake of serotonin into cardiomyocytes, and could be partly mediated by a mitochondrial dysfunction [14]. Polymorphisms in the *5-HT2A *receptor gene have been linked to receptor function [15]. A *5-HTT *gene polymorphism located in the promoter region has been associated with gene expression and an increased uptake of 5-HT in platelets [16]. Due to the central role of serotonin in brain function these gene variants have been extensively studied in neurological and psychiatric traits, but little is known about their role in cardiac hypertrophy [17].

Angiotensin II is formed from angiotensin I by the action of the angiotensin-II converting enzyme (ACE). Ang is a potent vasoconstrictor, but also modulates cardiac hypertrophy [18]. The pharmacological blockade of ACE reduced the hypertrophy secondary to myocardial infarction and hypertension [19]. Polymorphisms in the genes encoding angiotensinogen (*AGT*), angiotensin-II converting enzyme (*ACE*), and angiotensin II type 1 receptor (*AT1R*) have been extensively studied in cardiovascular diseases, including LVH [4,20,21]. The *ACE *insertion/deletion (I/D) variant was related with the extent of HCM in patients with sarcomeric mutations [22,23]. A common single nucleotide polymorphism (SNP) in the 3' untranslated region (UTR) of *AT1R *(1166 A/C) was associated with hypertension and coronary artery stenosis and vasoconstriction [24-26]. This SNP could also modulate the phenotype in patients with HCM [27].

Considering the role of the serotonin and angiotensin systems in cardiac hypertrophy, we hypothesized that DNA variants in the *5-HT2A*, *5-HTT*, *AGT*, *ACE*, and *AT1R *genes could influence the risk for LVH. To investigate this association, we genotyped patients with LVH and healthy controls for DNA polymorphisms at these genes. We also determined the effect of these gene polymorphisms on onset age and the extent of the hypertrophy.

## Methods

### Patients and controls

This study was part of a research project designated to analyse the association of DNA-variants to HCM-risk. In the period 1999-2009, a total of 245 non-related patients were recruited through the Cardiology Departments of Hospital Universitario Central Asturias (HUCA) and Hospital Universitario Valdecilla-Santander. The existence of cardiac hypertrophy was suspected on the basis of clinical manifestations (exertional dyspnea, palpitations, angina, or syncope). In all the patients, we used two-dimensional echocardiography to determine the interventricular septal thickness (IVS) by measuring in diastole at the level of the left ventricle minor axis [28]. The posterior wall thickness (PWT) was also measured, and the left ventricular wall thickness (LVWT) calculated as the sum of IVS and PWT.

Table 1 summarizes the main characteristics of patients. All them fulfilled the next inclusion criteria: they had an interventricular septum (IVS) > 13 mm, and the hypertrophy was not secondary to other cardiac diseases capable of producing LVH (such as hypertension, valvular disease, and myocardial infarction). Patients with relatives who had also been diagnosed with HCM and/or sudden cardiac death (SCD) were classified as familial cases. In apparently sporadic cases, we performed electro and echocardiographic examination to their parents when they were available for the study.

**Table 1:** Main characteristics of the patients with HCM and hypertensive LVH

	Total HCM (n = 245)	Familial HCM (n = 105; 43%)	Sporadic HCM* (n = 140; 57%)	Hypertensive LVH (n = 145)
				

Mean age at Diagnosis (years)	46 ± 13	37 ± 18	43 ± 19	58 ± 17
Range	8-76	8-72	21-76	35-75

Male	144 (59%)	68 (65%)	76 (56%)	(59%)

**Mean BMI**				
Male	27 ± 3	26 ± 3	27 ± 4	28 ± 4
Female	26 ± 4	25 ± 3	26 ± 3	28 ± 5

Mean IVS#	20 ± 5	22 ± 6	18 ± 7	15 ± 5
Mean PWT#	13 ± 5	14 ± 5	11 ± 6	10 ± 6
Mean LVWT#	34 ± 6	36 ± 6	30 ± 6	26 ± 6

Dyspnea	168 (69%)	78 (74%)	90 (64%)	30%
				
**NYHA index#**				
Class I-II	120 (49%)	49 (47%)	71 (51%)	85%
Class III-IV	48 (20%)	29 (28%)	19 (14%)	15%

Angina	96 (39%)	53 (50%)	43 (31%)	16%

Syncope	48 (20%)	25 (24%)	23 (16%)	6%

Atrial fibrillation	47 (19%)	23 (22%)	24 (17%)	15%

Arrhythmia (Holter monitoring)	55 (22%)	21 (20%)	34 (24%)	18%

LVOT > 30 mm Hg#	72 (29%)	34 (32%)	38 (27%)	30%

				

**Sarcomeric mutations**	40 (16%)	30 (29%)	10 (7%)	ND
*MYH7*	12 (5%)	11 (10%)	1(< 1%)	
*MYBPC3*	23 (9%)	16 (15%)	7(5%)	
*TNNT2*	4 (2%)	2 (2%)	2 (1%)	
*TPM1*	1 (< 1%)	1 (1%)	0	

* In 45 patients none of the parents were studied to exclude the presence of asymptomatic LVH.

# IVS: interventricular septum; PWT: posterior wall thickness; LVWT: left ventricular wall thickness; NYHA: New York Heart Association functional class; LVOT: left ventricular outflow tract gradient.

The presence of sarcomeric mutations was not determined (ND) in the hypertensive-LVH patients.

A second group of patients was composed by 145 non-related hypertensives with LVH (59% male; mean age at diagnosis, 58 ± 17 years; mean IVS = 15 ± 5 mm). The controls were a total of 300 healthy individuals aged 20 to 75 years (mean age 51 ± 17; 54% male), recruited through the Blood Bank and the Cardiology Department of HUCA. They did not have symptoms of cardiovascular diseases, but none was echocardiographically evaluated to exclude the presence of asymptomatic LVH. A total of 150 of these controls were examined through electrocardiography to exclude the existence of cardiac diseases. All the patients and controls were Caucasians from the Northern Spain regions of Asturias and Cantabria, and gave their informed consent to participate in the study, approved by the Ethical Committee of Hospital Central Asturias.

### Sarcomeric gene mutations

Because HCM is commonly linked to mutations in cardiac sarcomeric genes, we determined the presence of mutations in the most frequently mutated genes in the 245 HCM-patients. The beta-myosin heavy chain (*MYH7*), cardiac troponin T (*TNNT2*), alpha-tropomyosin (*TPM1*), cardiac troponin I (*TNNI3*), and myosin binding protein C3 (*MYBPC3*) genes were sequenced as reported [29,30].

### Genotyping of the serotonin and angiotensin system polymorphisms

Two types of polymorphisms were analysed: insertion/deletion (*ACE *and *5-HTT*), and SNPs (*AGT*, *AT1R*, and *5-HT2A*). The genomic DNA of patients and controls was polymerase chain reaction (PCR) amplified (32 cycles) with specific primers, and the reactions were directly electrophoresed on 3% agarose gels (insertion/deletion alleles) or after digestion with a restriction enzyme (SNPs), as reported [31-33]. Alleles in the coding region were numbered following the standard nomenclature [34]. The reference numbers for the five polymorphisms were: rs699 (*AGT*, c.803 T/C); rs6313 (*5-HT2A*, c.102 T/C); rs5186 (*AT1R*, c.1166 A/C); rs4646994 (*ACE*, intron 16 I/D); rs4795541 (*5-HTT*, promoter l/s) (see the *Ensembl *database for the definition of these gene variants; http://www.ensembl.org). In the additional table 1 we summarized the primer sequences and genotyping conditions for the five polymorphisms.

### Statistical analysis

The Kolmogorov-Smirnov was used to determine whether the continuous variables followed a normal distribution. The mean values for variables that were normally distributed were compared between the different groups through the ANOVA. Allele and genotype frequencies between patients and controls were compared through a χ^2 ^test. Odds ratios (ORs) and their 95% confidence intervals (CIs) were also calculated. The SPSS package (v. 11.0) was used for all the statistical analysis. A p < 0.05 was considered statistically significant. Power calculation at p = 0.05 and p = 0.01 was performed for all the significant genetic associations with an online program http://statpages.org/proppowr.html.

## Results

Table 1 summarizes the main characteristics of the patients. A total of 75 of the HCM patients had at least one relative who was also affected by HCM or had suffered SCD. The remaining 170 patients did not have a family history of the disease, but the existence of asymptomatic relatives with HCM could not be excluded. In 90 of these patients we performed electro and echocardiographic examination to both parents, and to only one parent in 35 cases. HCM was also found in the father or the mother of 30 of these 125 HCM-patients, that could thus be regarded as familial cases. In 45 patients, none of the parents were available for study.

A total of 40 of the 245 HCM-patients had a mutation in the *MYH7*, *MYBPC3*, *TPM1*, *TNNI3*, or *TNNT2 *genes (Additional table 2). Sarcomeric mutations were more frequent in patients with familial HCM compared to apparently sporadic cases (30% vs. 7%). The genotyping of the *5-HT2A*, *5-HTT*, *AGT*, *ACE*, and *AT1R *polymorphisms showed a significantly higher frequency of carriers of the *AT1R *C allele (AC+CC genotypes) in the HCM patients without sarcomeric gene mutations compared to the healthy controls (p = 0.015; OR = 1.56; 95% CI = 1.09-2.23) (Table 2). The difference was no significant when the Bonferroni's correction was applied p < 0.01). The sample size (205 patients and 300 controls) was enough to reach a power of 75% at a p = 0.05 (for a power of 80%, a total of 225 patients and 338 controls should be required at a p = 0.05, and 336 patients and 504 controls at a p = 0.01). The frequency of *AT1R *C-carriers did not differ between hypertensives with LVH and controls (50% vs. 47%).

**Table 2:** Genotype and allele frequencies for the five polymorphisms in patients and healthy controls

Polymorphism	HCM* N=205	Hypertensive LVH N = 145	Controls N = 300
*5-HT2A *(c.102 T/C) Rs6313			

TT	45 (22%)	24 (17%)	60 (20%)

TC	105 (51%)	79 (54%)	149 (50%

CC	55 (27%)	42 (29%)	91 (30%)

T	0.47	0.43	0.45

C	0.53	0.57	0.55

			

*5-HTT *(l/s) Rs4795541			

ll	72 (35%)	48 (33%)	91 (30%)

ls	102 (50%)	71 (49%)	147 (49%)

ss	31 (15%)	26 (18%)	62 (21%)

l	0.60	0.58	0.55

s	0.40	0.42	0.45

			

*ACE *(I/D) Rs4646994			

DD	72 (35%)	54 (37%)	119 (40%)

ID	100 (48%)	68 (45%)	135 (45%)

II	35 (17%)	23 (15%)	46 (15%)

D	0.59	0.61	0.62

I	0.41	0.39	0.38

			

*AGT *(c.803 T/C) Rs699			

MM	64 (31%)	54 (37%)	95 (32%)

MT	100 (49%)	68 (48%)	145 (48%)

TT	41 (19%)	22 (15%)	60 (20%)

M	0.55	0.61	0.56

T	0.45	0.39	0.44

			

*AT1R *(c.1166 A/C)#			
Rs5182			

AA	84 (41%)	72 (50%)	156 (53%)

AC	94 (46%)	60 (41%)	114 (37%)

CC	27 (13%)	13 (9%)	30 (10%)

A	0.64	0.70	0.71

C	0.36	0.30	0.29

*Patients without sarcomeric mutations.

# HCM vs. controls: p = 0.015; OR = 1.56 (95%CI = 1.09-2.23); AC + CC HCM patients vs. controls.

We examined the difference for the main characteristics between the *5-HT2A*, *5-HTT*, *AGT*, *ACE*, and *AT1R *genotypes in the 205 patients without sarcomeric mutations. We found a higher frequency of familial cases among *AT1R *C-carriers (p = 0.02), and this could reflect a predisposition to develop familial cardiac hypertrophy linked to these genes. We also found a higher mean IVS and LVWT among patients who were *AT1R *CC/AC compared to AA in both HCM groups, with and without sarcomeric mutations (Table 3). The *AT1R *genotype did not modify the mean IVS and LVWT among the hypertensive patients.

**Table 3:** Mean (± Standard deviation) interventricular septum, posterior wall thickness, left ventricular wall thickness, age at the diagnostis and body mass index values, and frequency of cases with affected relatives, according to the *AT1R *genotype in the 205 HCM-patients without sarcomeric mutations, the 40 patients with a sarcomeric mutation, and the 145 patients with hypertensive LVH

	IVS (mm)	PWT (mm)	LVWT (mm)	Age (years)	BMI	Familial HCM#
**HCM-No mutation^1^**						
CC (n = 27)	21 ± 4	13 ± 3	34 ± 5	49 ± 18	26 ± 5	10 (37%)
AC (n = 94)	21 ± 5	13 ± 4	33 ± 4	46 ± 18	27 ± 4	40 (43%)
AA (n = 84)	19 ± 5	13 ± 4	32 ± 4	48 ± 16	27 ± 5	25 (30%)

**HCM-Mutation^2^**						
CC (n = 5)	23 ± 4	16 ± 3	39 ± 4	38 ± 4	21 ± 4	4 (80%)
AC (n = 14)	22 ± 5	14 ± 5	35 ± 5	36 ± 5	21 ± 5	12 (86%)
AA (n = 21)	18 ± 5	14 ± 4	31 ± 5	45 ± 5	20 ± 5	14 (67%)

**Hypertensive-LVH**						
CC (n = 13)	16 ± 4	10 ± 5	25 ± 5	60 ± 8	28 ± 2	ND
AC (n = 60)	16 ± 3	9 ± 4	25 ± 4	58 ± 7	27 ± 2	ND
AA (n = 72)	15 ± 2	10 ± 5	24 ± 4	59 ± 9	28 ± 3	ND

# We did not determine (ND) the existence of a family history of LVH in the hypertensive-LVH group.

^**1 **^P = 0.016, IVS CC + AC vs. AA.

^**2 **^P = 0.017, IVS CC + AC vs. AA.

Several DNA polymorphisms in the angiotensin system genes have been proposed as modifiers of the phenotype in families with sarcomeric mutations. In our study, patients with a sarcomere mutation (n = 40) who were *AT1R *CC/AC had higher mean IVS and LVWT, and lower mean onset age compared to *AT1R *AA. In addition, *AT1R *C - carriers had a higher frequency of familial cases (table 3). However, these differences did not reach statistical significance, probably because they were based on only 40 index patients with a sarcomeric mutation. Because *MYH7 *mutations have been associated with more severe forms of HCM compared to *MYBPC3*, we also compared the effect of the *AT1R *SNP according to the mutated gene. We studied 19 mutation carriers from the 12 families with a *MYH7*-mutation, and 64 mutation carriers from the 23 families with a *MYBPC3*-mutation (Additional table 2). We found a total of 48 *AT1R *C carriers, 9 in the *MYH7 *and 39 in the *MYBPC3 *groups, and the mean LVWT was higher among these *AT1R *C carriers compared to *AT1R *AA in the two groups, although the difference did not reach statistical significance (p = 0.053).

## Discussion

In this study we genotyped 245 HCM-patients and 300 healthy controls for 5 polymorphisms in five candidate genes of the angiotensin and serotonin systems. We identified an HCM-causative mutation in one of the five most commonly mutated sarcomeric genes (*MYH7*, *MYBPC3*, *TPM1*, *TNNI3*, or *TNNT2*) in 40 cases, but we cannot exclude that other patients harbour mutations in any of the other genes that have been linked to HCM. However, we think this would affect a reduced number of cases because the five sarcomeric genes represent > 90% of the mutations found in HCM-patients (see the cardiogenomics database; http://www.cardiogenomics.org). The frequency of patients with a sarcomeric mutation (16.3%) was lower than the frequency previously reported in our population (27%). This could be partly attributed to a lower frequency of cases with affected relatives and a mean higher onset age for the patients in this study, compared to previous reports [29,30].

We found a significantly higher frequency of *AT1R *C-carriers among patients negative for sarcomeric mutations, compared to healthy controls. This could represent a predisposition to develop HCM among individuals with this *AT1R *allele, although the OR was relatively low (1.56) in this group of patients and 41% of the 205 patients without a myofilament mutation were non-carriers of this allele. The *AT1R *1166 C has been associated with the risk for several cardiovascular traits, including hypertension, coronary artery vasoconstriction, and coronary artery disease. Some authors did not find a significant association between this allele and the risk for HCM [35]. However, in these studies the patients were not selected by the presence/absence of sarcomeric gene mutations, and this could result in a non-significant association if patients with a causative HCM mutation were included in the study. In fact, the *AT1R *frequencies did not differ between our patients with sarcomeric mutations and controls. Moreover, if we compared the *AT1R *genotype frequencies between all the HCM patients (n = 245) and the controls (n = 300), no significant difference was found for 1166 C carriers (p = 0.06). A total of 30 patients without sarcomeric mutations had a positive family history of HCM. It is possible that the frequency of *AT1R *C carriers was also significantly higher among these affected relatives. However, this information was not available because these individuals were not genotyped for the *AT1R *polymorphism.

The *AT1R *SNP has also been proposed as a modifier of the clinical phenotype in HCM [4-6]. In their analysis of 389 HCM-patients (45% with a family history of HCM and/or SCD), Perkins et al. reported a lower mean age at diagnosis among *AT1R *CC compared to *AT1R *AA (37.9 vs. 43.2 years, respectively). We did not find significantly different mean onset ages between the *AT1R *genotypes, although patients with a sarcomeric mutation and *AT1R *C-carriers had a lower mean onset age. This suggested that the *AT1R *genotype could be a modifier of the onset age among patients with a causative sarcomeric mutation. Perkins et al. also reported higher mean left ventricular wall thickness and a higher frequency of severe hypertrophy (> 30 mm) among *AT1R *CC patients. This association with the extent of LVH was also reported by others [27]. We also found a higher mean LVWT among *AT1R *C-carriers in both, patients with and without sarcomeric mutation. Moreover, this allele was associated with higher LVWT in patients with *MYH7 *and *MYBPC3 *mutations. This suggested that the *AT1R *genotype could be a modifier of the extent of the hypertrophy in our population, in patients with and without sarcomeric mutations. The role of the *AT1R *SNP as a modifier of the phenotype was also supported by the finding of a higher frequency of familial HCM among patients with sarcomeric mutations and 1166 C-carriers. This could be the consequence of a more severe phenotype among *AT1R *C-carriers, resulting in a higher penetrance of the sarcomeric mutation among carriers of this *AT1R *allele. However, our definition of familial HCM was incomplete because in 19% of our patients who did not have a family history of the disease we could not exclude the presence of asymptomatic LVH in their parents. It is thus possible that the frequency of familial cases was higher than estimated in our patients, and this could affect the results.

The *AT1R *1166A/C (SNP rs5186) is in the 3' UTR region, in a sequence that binds microRNA (miRNA) -155. MiRNAs are small (approximately 22 nucleotides long) non-coding RNAs that bind to sequences in the 3' UTRs of mRNAs by complementary base-pairing, and repress mRNA post-transcriptionally. The + 1166 C-allele determines the interruption of the base-pairing complementarity with miR-155, and this resulted in the increased translation of *AT1R *compared to the mRNA containing 1166 A [36]. Both, *AT1R *and miR-155, are abundantly expressed in the same cell types (e.g. VSMCs and endothelial). The regulation of *AT1R *by miR-155 and the differential binding of this miRNA to mRNAs with 1166 A or C provided a mechanism by which this SNP could lead to a heterogeneous AT1R expression and cardiovascular risk. Although a direct effect of this SNP on AT1R expression could explain its association to cardiac hypertrophy and other cardiovascular disorders, we cannot exclude that this association was a consequence to its linkage disequilibrium with other *AT1R *variants. For this gene two main haplotype blocks have been identified, one defined by markers in the promoter region and the other by SNPs rs5182 and rs5186 in the 3' region [37,38]. A resequencing of the AT1R in patients carrying the C-allele should be necessary to identify other variants that could be linked to the risk for cardiac hypertrophy. In addition, the pharmacological blockade of angiotensin II receptors has been shown to reduce LVH, and could be useful to treat this disease [39]. A significant association between the *AT1R *1166 A/C SNP and LVH change during antihypertensive treatment with AT1R antagonists has been reported [40]. In this context, it should be interesting to evaluate the effect of the *AT1R *genotypes on the response to AT1R antagonists in patients with HCM.

Finally, our study has some limitations that could affect the results. The association between the *AT1R *SNP and HCM was significant (p = 0.015), but the OR for allele C-carriers was 1.56 and the lower limit of CI (1.09) was close to 1. Although the association was plausible considering the statistical power, it should be replicated in larger cohorts and from different populations. As discussed above, the five sarcomeric genes analysed in our patients would represent > 90% of the mutated genes in HCM patients. However, mutations in more than 12 genes have been found in HCM cases and some of the 205 patients could be included as carriers of a myofilament mutation if all these genes were studied. Third, we found a significant association between the *AT1R *and familial HCM in patients without sarcomeric gene mutations, but our classification of familial/sporadic cases was incomplete because we did not perform ECG or echocardiographic examination to all the first degree relatives of our patients. It is thus possible that some patients had relatives with asymptomatic LVH, and could thus be classified as familial cases.

## Conclusions

The *AT1R *1166 A/C polymorphism was associated with HCM in patients without sarcomeric gene mutations. In addition, the frequency of familial hypertrophy was higher among carriers of this allele, and we also found a trend toward higher left ventricular thickness among these 1166 C-carries. Our work suggested that the *AT1R *gene variation could contribute to the risk of developing cardiac hypertrophy, being also a modifier of the phenotype.

## Conflict of interests Disclosure

The authors declare that they have no competing interests.

## Authors' contributions

EC designated the study, performed the statistical analysis, and wrote the manuscript. JRR, MM, JRB, FO and CM, recruited the patients/controls and obtained the clinical and anthropometric data. EC, MP, CG, MGC, BT, AIC, MD, BM, and VA performed all the genetic studies. All the authors have read and approved the final manuscript.

## Supplementary Material

Additional file 1 **Additional table 1**. Primers used to amplify the five polymorphic sites, annealing temperature, restriction enzymes to digest the PCR-products, and size of the alleles. Primers were derived from the reference sequences for the five genes in the Ensembl database http://www.ensembl.org: *ACE*, ENSG00000159640; *5-HTT*, ENSG00000108576; *AGT*, ENST00000366667; *5-HT2A*, ENST00000378688; *AT1R*, ENST00000349243. Click here for file

Additional file 2 **Additional table 2**. Summary of the 40 HCM cases with sarcomeric gene mutations. In each family, we indicated the mutation, the number of mutation carriers in the family who were *AT1R *CC/AC or AA, and the mean onset age and mean LVWT according to the *AT1R *genotype. Click here for file

## References

[B1] Sadoshima J Izumo S The cellular and molecular response of cardiac myocytes to mechanical stress Annu Rev Physiol 1997 59 551 571 10.1146/annurev.physiol.59.1.551 9074777

[B2] Lorell BH Carabello BA Left ventricular hypertrophy: pathogenesis, detection, and prognosis Circulation 2000 102 470 479 10908222 10.1161/01.cir.102.4.470

[B3] Bos JM Towbin JA Ackerman MJ Diagnostic prognostic, and therapeutic implications of genetic testing for hypertrophic cardiomyopathy J Am Coll Cardiol 2009 54 201 211 10.1016/j.jacc.2009.02.075 19589432

[B4] Bleumink GS Schut AF Sturkenboom MC Deckers JW van Duijn CM Stricker BH Genetic polymorphisms and heart failure Genet Med 2004 6 465 474 10.1097/01.GIM.0000144061.70494.95 15545741

[B5] Keren A Syrris P McKenna WJ Hypertrophic cardiomyopathy: the genetic determinants of clinical disease expression Nat Clin Pract Cardiovasc Med 2008 5 158 168 10.1038/ncpcardio1110 18227814

[B6] Perkins MJ Van Driest SL Ellsworth EG Will ML Gersh BJ Ommen SR Ackerman MJ Gene-specific modifying effects of pro-LVH polymorphisms involving the renin-angiotensin-aldosterone system among 389 unrelated patients with hypertrophic cardiomyopathy Eur Heart J 2005 26 2457 2462 10.1093/eurheartj/ehi438 16087648

[B7] Heineke J Molkentin JD Regulation of cardiac hypertrophy by intracellular signalling pathways Nat Rev Mol Cell Biol 2006 7 589 600 10.1038/nrm1983 16936699

[B8] Jaffré F Bonnin P Callebert J Debbabi H Setola V Doly S Monassier L Mettauer B Blaxall BC Launay JM Maroteaux L Serotonin and angiotensin receptors in cardiac fibroblasts coregulate adrenergic-dependent cardiac hypertrophy Circ Res 2009 104 113 123 10.1161/CIRCRESAHA.108.180976 19023134

[B9] Jaffré F Callebert J Sarre A Etienne N Nebigil CG Launay JM Maroteaux L Monassier L Involvement of the serotonin 5-HT2B receptor in cardiac hypertrophy linked to sympathetic stimulation: control of interleukin-6, interleukin-1 beta, and tumor necrosis factor-alpha cytokine production by ventricular fibroblasts Circulation 2004 110 969 974 10.1161/01.CIR.0000139856.20505.57 15302781

[B10] Sano M Fukuda K Kodama H Pan J Saito M Matsuzaki J Takahashi T Makino S Kato T Ogawa S Interleukin-6 family of cytokines mediate angiotensin II-induced cardiac hypertrophy in rodent cardiomyocytes J Biol Chem 2000 275 29717 29723 10.1074/jbc.M003128200 10843995

[B11] Cerrito F Lazzaro MP Gaudio E Arminio P Aloisi G 5HT2-receptors and serotonin release their role in human platelet aggregation Life Sci 1993 53 209 215 10.1016/0024-3205(93)90671-O 8321084

[B12] Eddahibi S Fabre V Boni C Martres MP Raffestin B Hamon M Adnot S Induction of serotonin transporter by hypoxia in pulmonary vascular smooth muscle cells. Relationship with the mitogenic action of serotonin Circ Res 1999 84 329 336 10024307 10.1161/01.res.84.3.329

[B13] Dorn GW Tepe NM Lorenz JN Koch WJ Liggett SB Low- and high-level transgenic expression of beta2-adrenergic receptors differentially affect cardiac hypertrophy and function in Galphaq-overexpressing mice Proc Natl Acad Sci USA 1999 96 6400 6405 10.1073/pnas.96.11.6400 10339599 PMC26893

[B14] Nebigil CG Maroteaux L Functional consequence of serotonin/5-HT2B receptor signaling in heart: role of mitochondria in transition between hypertrophy and heart failure? Circulation 2003 108 902 908 10.1161/01.CIR.0000081520.25714.D9 12925446

[B15] Arranz M Collier D Sodhi M Ball D Roberts G Price J Sham P Kerwin R Association between clozapine response and allelic variation in 5-HT2A receptor gene Lancet 1995 346 281 282 10.1016/S0140-6736(95)92168-0 7630250

[B16] Greenberg BD Tolliver TJ Huang S-H Li Q Bengel D Murphy DL Genetic variation in the serotonin transporter promoter region affects serotonin uptake in human blood platelets Am J MedGenet 1999 88 83 87 10.1002/(SICI)1096-8628(19990205)88:1<83::AID-AJMG15>3.0.CO;2-0 10050973

[B17] D'Souza UM Craig IW Functional genetic polymorphisms in serotonin and dopamine gene systems and their significance in behavioural disorders Prog Brain Res 2008 172 73 98 full_text 18772028 10.1016/S0079-6123(08)00904-7

[B18] Harrap SB Dominiczak AF Fraser R Lever AF Morton JJ Foy CJ Watt GCM Plasma angiotensin II, predisposition to hypertension, and left ventricular size in healthy young adults Circulation 1996 93 1148 1154 8653835 10.1161/01.cir.93.6.1148

[B19] Johnson DB Foster RE Barilla F Blackwell GG Roney M Stanley AWH Jr Kirk K Orr RA van der Geest RJ Reiber JHC Dell'Italia LJ. Angiotensin-converting enzyme inhibitor therapy affects left ventricular mass in patients with ejection fraction < 40% after acute myocardial infarction J Am Coll Cardiol 1997 29 49 54 10.1016/S0735-1097(96)00451-2 8996294

[B20] Wang JG Staessen JA Genetic polymorphisms in the renin-angiotensin system: relevance for susceptibility to cardiovascular disease Eur J Pharmacol 2000 410 289 302 10.1016/S0014-2999(00)00822-0 11134677

[B21] Hebert PR Foody JM Hennekens CH The renin-angiotensin system: the role of inhibitors, blockers, and genetic polymorphisms in the treatment and prevention of heart failure Curr Vasc Pharmacol 2003 1 33 39 10.2174/1570161033386655 15320851

[B22] Lechin M Quinones MA Omran A Hill R Yu QT Rakowski H Wigle D Liew CC Sole M Roberts R Marian AJ Angiotensin I converting enzyme genotypes and left ventricular hypertrophy in patients with hypertrophic cardiomyopathy Circulation 1995 92 1808 1812 7671365 10.1161/01.cir.92.7.1808

[B23] Tesson F Dufour C Moolman JC Carrier L Al-Mahdawi S Chojnowska L Dubourg O Soubrier F Brink P Komajda M Guicheney P Schwartz K Feingold J The influence of the angiotensin I converting enzyme genotype in familial hypertrophic cardiomyopathy varies with the disease gene mutation J Mol Cell Cardiol 1997 29 831 838 10.1006/jmcc.1996.0332 9140839

[B24] Benetos A Gautier S Ricard S Topouchian J Asmar R Poirier O Larosa E Guize L Safar M Soubrier F Cambien F Influence of angiotensin-converting enzyme and angiotensin II type 1 receptor gene polymorphisms on aortic stiffness in normotensive and hypertensive patients Circulation 1996 94 698 703 8772690 10.1161/01.cir.94.4.698

[B25] Nakauchi Y Suehiro T Yamamoto M Yasuoka N Arii K Kumon Y Hamashige N Hashimoto K Significance of angiotensin I-converting enzyme and angiotensin II type 1 receptor gene polymorphisms as risk factors for coronary heart disease Atherosclerosis 1996 125 161 169 10.1016/0021-9150(96)05866-2 8842348

[B26] Amant C Hamon M Bauters C Richard F Helbecque N McFadden EP Escudero X Lablanche JM Amouyel P Bertrand ME The angiotensin II type 1 receptor gene polymorphism is associated with coronary artery vasoconstriction J Am Coll Cardiol 1997 29 486 490 10.1016/S0735-1097(96)00535-9 9060882

[B27] Osterop AP Kofflard MJ Sandkuijl LA ten Cate FJ Krams R Schalekamp MA Danser AH T1 receptor A/C1166 polymorphism contributes to cardiac hypertrophy in subjects with hypertrophic cardiomyopathy Hypertension 1998 32 825 830 9822439 10.1161/01.hyp.32.5.825

[B28] Lang RM Bierig M Devereux RB Flachskampf FA Foster E Pellikka PA Picard MH Roman MJ Seward J Shanewise JS Solomon SD Spencer KT Sutton MS Stewart WJ Recommendations for chamber quantification: a report from the American Society of Echocardiography's Guidelines and Standards Committee and the Chamber Quantification Writing Group, developed in conjunction with the European Association of Echocardiography, a branch of the European Society of Cardiology J Am Soc Echocardiogr 2005 18 1440 1463 10.1016/j.echo.2005.10.005 16376782

[B29] García-Castro M Reguero JR Batalla A Díaz-Molina B González P Alvarez V Cortina A Cubero GI Coto E Hypertrophic cardiomyopathy: low frequency of mutations in the beta-myosin heavy chain (MYH7) and cardiac troponin T (TNNT2) genes among Spanish patients Clin Chem 2003 49 1279 1285 10.1373/49.8.1279 12881443

[B30] García-Castro M Coto E Reguero JR Berrazueta JR Alvarez V Alonso B Sainz R Martín M Morís C Mutations in sarcomeric genes MYH7, MYBPC3, TNNT2, TNNI3, and TPM1 in patients with hypertrophic cardiomyopathy Rev Esp Cardiol 2009 62 48 56 10.1016/S1885-5857(09)71513-0 19150014

[B31] Alvarez R Reguero JR Batalla A Iglesias-Cubero G Cortina A Alvarez V Coto E Angiotensin-converting enzyme and angiotensin II receptor 1 polymorphisms: association with early coronary disease Cardiovasc Res 1998 40 375 379 10.1016/S0008-6363(98)00179-5 9893731

[B32] Coto E Reguero JR Alvarez V Morales B Batalla A González P Martín M García-Castro M Iglesias-Cubero G Cortina A 5-Hydroxytryptamine 5-HT2A receptor and 5-hydroxytryptamine transporter polymorphisms in acute myocardial infarction Clin Sci (Lond) 2003 104 241 245 10.1042/CS20020246 12605580

[B33] Coto E Rodrigo L Alvarez R Fuentes D Rodríguez M Menéndez LG Ciriza C González P Alvarez V Variation at the Angiotensin-converting enzyme and endothelial nitric oxide synthase genes is associated with the risk of esophageal varices among patients with alcoholic cirrhosis J Cardiovasc Pharmacol 2001 38 833 839 10.1097/00005344-200112000-00004 11707686

[B34] den Dunnen JT Antonarakis SE Mutation nomenclature extensions and suggestions to describe complex mutations: a discussion Hum Mutat 2000 15 7 12 10.1002/(SICI)1098-1004(200001)15:1<7::AID-HUMU4>3.0.CO;2-N 10612815

[B35] Brugada R Kelsey W Lechin M Zhao G Yu QT Zoghbi W Quinones M Elstein E Omran A Rakowski H Wigle D Liew CC Sole M Roberts R Marian AJ Role of candidate modifier genes on the phenotypic expression of hypertrophy in patients with hypertrophic cardiomyopathy J Invest Med 1997 45 542 551 9444881

[B36] Martin MM Buckenberger JA Jiang J Malana GE Nuovo GJ Chotani M Feldman DS Schmittgen TD Elton TS The human angiotensin II type 1 receptor + 1166 A/C polymorphism attenuates microrna-155 binding J Biol Chem 2007 282 24262 24269 10.1074/jbc.M701050200 17588946 PMC2413065

[B37] Su X Lee L Li X Lv J Hu Y Zhan S Cao W Mei L Tang YM Wang D Krauss RM Taylor KD Rotter JI Yang H Association between angiotensinogen, angiotensin II receptor genes, and blood pressure response to an angiotensin-converting enzyme inhibitor Circulation 2007 115 725 732 10.1161/CIRCULATIONAHA.106.642058 17261659

[B38] Abdollahi MR Lewis RM Gaunt TR Cumming DV Rodriguez S Rose-Zerilli M Collins AR Syddall HE Howell WM Cooper C Godfrey KM Cameron IT Day IN Quantitated transcript haplotypes (QTH) of AGTR1, reduced abundance of mRNA haplotypes containing 1166C (rs5186:A > C), and relevance to metabolic syndrome traits Hum Mutat 2007 28 365 373 10.1002/humu.20454 17211857

[B39] Verdecchia P Sleight P Mancia G Fagard R Trimarco B Schmieder RE Kim JH Jennings G Jansky P Chen JH Liu L Gao P Probstfield J Teo K Yusuf S ONTARGET/TRANSCEND Investigators. Effects of telmisartan, ramipril, and their combination on left ventricular hypertrophy in individuals at high vascular risk in the Ongoing Telmisartan Alone and in Combination With Ramipril Global End Point Trial and the Telmisartan Randomized Assessment Study in ACE Intolerant Subjects With Cardiovascular Disease Circulation 2009 120 1380 1389 10.1161/CIRCULATIONAHA.109.865774 19770395

[B40] Kurland L Melhus H Karlsson J Kahan T Malmqvist K Ohman P Nyström F Hägg A Lind L Polymorphisms in the angiotensinogen and angiotensin II type 1 receptor gene are related to change in left ventricular mass during antihypertensive treatment: results from the Swedish Irbesartan Left Ventricular Hypertrophy Investigation versus Atenolol (SILVHIA) trial J Hypertens 2002 20 657 663 10.1097/00004872-200204000-00023 11910301

