# Supplementary material for: Functional polymorphisms in genes of the Angiotensin and Serotonin systems and risk of hypertrophic cardiomyopathy: AT1R as a potential modifier
Source: J Transl Med. 2010 Jul 1;8:64. doi: 10.1186/1479-5876-8-64 (PMC2907326; doi:10.1186/1479-5876-8-64)
Supplement: Additional file 1 — Additional table 1. Primers used to amplify the five polymorphic sites, annealing temperature, restriction enzymes to digest the PCR-products, and size of the alleles. Primers were derived from the reference sequences for the five genes in the Ensembl database http://www.ensembl.org: ACE, ENSG00000159640; 5-HTT, ENSG00000108576; AGT, ENST00000366667; 5-HT2A, ENST00000378688; AT1R, ENST00000349243. [file 1479-5876-8-64-S1.DOC]

**Additional** **Table 1.** Primers used to amplify the five polymorphic sites, annealing temperature, restriction enzymes to digest the PCR-products, and size of the alleles.

Primers were derived from the reference sequences for the five genes in the Ensembl database ([www.ensembl.org](http://www.ensembl.org/) ): *ACE*, ENSG00000159640; *5-HTT*, ENSG00000108576; *AGT*, ENST00000366667; *5-HT2A*, ENST00000378688; *AT1R*, ENST00000349243.

| Polymorphism | PCR-primers | Annealing  Temp. | Size  (bp) | Restriction  enzyme | Alleles size  (bp) |
| --- | --- | --- | --- | --- | --- |
| *5-HT2A*  c.102 T/C | Fwd. TCTGCTACAAGTTCTGGCTT  Rvs. CTGCAGCTTTTTCTCTAGGG | 62ºC | 342 | *MspI* | T= 342  C= 216+126 |
| *5-HTT*  Promoter  l/s | Fwd. GGCGTTGCCGCTCTGAATTGC  Rvs.GAGGGACTGAGCTGGACAACCCAC | 65ºC | 185/  240 | - | l= 240  s= 185 |
| *AGT*  c.803 T/C | Fwd. GATGCGCACAAGGTCCTG  Rvs. CAGGGTGCTGTCCACACTGGCTCGC | 62ºC | 303 | *Bst*UI | M=303  T=279+24 |
| *ACE*  I/D | Fwd. CTGGAGACCACTCCCATCCTTTCT  Rvs. ATGTGGCCATCACATTCGTCAGAT | 58ºC | 390/  190 | - | I =390  D = 190 |
| *AT1R*  c.1166 A/C | Fwd. GCAG CACTTCACTACCAAATGAT  Rvs. TGTTCTTCGAGCAGCCGT | 58ºC | 176 | *BclI* | C=176  A=156+20 |
