# Supplementary material for: Functional polymorphisms in genes of the Angiotensin and Serotonin systems and risk of hypertrophic cardiomyopathy: AT1R as a potential modifier
Source: J Transl Med. 2010 Jul 1;8:64. doi: 10.1186/1479-5876-8-64 (PMC2907326; doi:10.1186/1479-5876-8-64)
Supplement: Additional file 2 — Additional table 2. Summary of the 40 HCM cases with sarcomeric gene mutations. In each family, we indicated the mutation, the number of mutation carriers in the family who were AT1R CC/AC or AA, and the mean onset age and mean LVWT according to the AT1R genotype. [file 1479-5876-8-64-S2.DOC]

**Additional table 2**. Summary of the 40 HCM cases with sarcomeric gene mutations. In each family, we indicated the mutation, the number of mutation carriers in the family who were *AT1R* CC/AC or AA, and the mean onset age and mean LVWT according to the *AT1R* genotype.

| Case /  family | Gene | Mutation# | Mutation Carriers  AT1R :  CC/AC + AA | Mean onset age  CC+AC / AA | Mean LVWT  CC+AC / AA |
| --- | --- | --- | --- | --- | --- |
| 123 | *MYBPC3* | G531R | 2 + 0 | 45 / 0 | 35 / 0 |
| 240 | *MYBPC3* | Q998E | 5 + 1 | 37 / 45 | 38 /33 |
| 41 | *MYBPC3* | A627V | 4 + 0 | 30 / 0 | 45 / 0 |
| 227 | *MYBPC3* | E542Q | 1 + 0 | 32 / 0 | 35 / 0 |
| 213 | *MYBPC3* | R891fs | 3 + 0 | 48 / 0 | 33 / 0 |
| 149 | *MYBPC3* | R726C | 1 + 0 | 68 / 0 | 36 / 0 |
| 92 | *MYBPC3* | M844fs | 9 + 3 | 43 / 48 | 28 / 27 |
| 103 | *MYBPC3* | R495W | 4 + 2 | 25 / 28 | 37 / 32 |
| 143 | *MYBPC3* | G263X | 1 + 0 | 39 / 0 | 39 / 0 |
| 77 | *MYBPC3* | A328fs | 1 + 2 | 30 / 33 | 41 / 39 |
| 99 | *MYBPC3* | R1022S | 2 + 1 | 31 / 30 | 39 / 37 |
| 238 | *MYBPC3* | E542Q | 0 + 1 | 0 / 57 | 0 / 31 |
| 242 | *MYBPC3* | E542Q | 0 + 1 | 0 / 51 | 0 / 29 |
| 239 | *MYBPC3* | E542Q | 1 + 1 | 50 / 45 | 31 / 29 |
| 212 | *MYBPC3* | Q404fs | 0 + 1 | 0 / 31 | 0 / 33 |
| 33 | *MYBPC3* | G532fs | 0 + 1 | 0 / 59 | 0 / 27 |
| 87 | *MYBPC3* | M844fs | 0 + 1 | 0 / 57 | 0 / 34 |
| 47 | *MYBPC3* | R1080H | 0 + 1 | 0 / 60 | 0 / 38 |
| 115 | *MYBPC3* | G263X | 2 + 3 | 33 / 42 | 35 / 32 |
| 134 | *MYBPC3* | Y237C | 2+ 2 | 34 / 39 | 50 / 39 |
| 16 | *MYBPC3* | V771M | 1+2 | 8 / 23 | 29 / 31 |
| 225 | *MYBPC3* | E542Q | 0+1 | 0 / 37 | 0 / 30 |
| 111 | *MYBPC3* | G263X | 0+1 | 0 / 41 | 0 / 32 |
|  | **Total**  ***MYBPC3*** | **N= 23** | **39 + 25** | **40 / 44** | **34 / 31** |
| 168 | *MYH7* | R870H | 1 + 1 | 49 / 42 | 39 / 37 |
| 160 | *MYH7* | A99T | 0 + 1 | 0 / 49 | 0 / 32 |
| 68 | *MYH7* | F247L | 1 + 1 | 42 / 35 | 30 / 34 |
| 159 | *MYH7* | I927F | 1 + 1 | 52 /58 | 42 / 28 |
| 29 | *MYH7* | R453C | 0 + 1 | 0 / 25 | 0 / 29 |
| 226 | *MYH7* | R663H | 1 + 2 | 44 / 51 | 41 / 32 |
| 136 | *MYH7* | R723G | 0 + 1 | 0 / 31 | 0 / 38 |
| 74 | *MYH7* | A583V | 1 + 0 | 34 /0 | 39 / 0 |
| 113 | *MYH7* | A583V | 1 + 2 | 16 / 35 | 39 / 35 |
| 193 | *MYH7* | P828S | 1 + 0 | 24 / 0 | 40 / 0 |
| 3 | *MYH7* | R787C | 1 + 0 | 51 / 0 | 32 / 0 |
| 26 | *MYH7* | V822M | 1 + 0 | 3 / 0 | 36 / 0 |
|  | **Total**  ***MYH7*** | **N=12** | **9 + 10** | **39 / 44** | **42 / 33** |

| 89 | *TNNT2* | R278C | 1 + 0 | 4 / 0 | 41 / 0 |
| --- | --- | --- | --- | --- | --- |
| 185 | *TNNT2* | R92Q | 1 + 0 | 32 / 0 | 33 / 0 |
| 85 | *TNNT2* | R92Q | 2 + 0 | 43 / 0 | 29 / 0 |
| 234 | *TNNT2* | R92Q | 1 + 0 | 37 / 0 | 32 / 0 |
| 13 | *TPM1* | D175N | 4 + 0 | 8 / 0 | 37 / 0 |

# See [www.cardiogenomics.org](http://www.cardiogenomics.org/) and ref. [29] for a complete description of the mutations.
